# Determination and Prediction of Available Energy in 13 Cereal Feed Ingredients for Growing Pigs

**DOI:** 10.3390/vetsci11120648

**Published:** 2024-12-13

**Authors:** Jinbiao Zhao, Xiaoming Song, Meiyu Yang, Ge Zhang, Ling Liu

**Affiliations:** State Key Laboratory of Animal Nutrition and Feeding, College of Animal Science and Technology, China Agricultural University, Beijing 100193, China; jinbiaozhao@cau.edu.cn (J.Z.);

**Keywords:** growing pigs, nutrient digestibility, available energy, prediction equations, cereals

## Abstract

This study was conducted to develop equations to predict the digestible (DE) and metabolizable energy (ME) for growing pigs by using the chemical compositions of five corn, two wheat and six rice samples. A total of 13 castrated boars were chosen and fed 13 diets formulated with different cereal feed ingredients according to a 13 × 6 Youden square design. The DE and ME contents, the ratio of ME to DE, and the nutrient digestibility among the 13 cereal feed ingredients were different (*p* < 0.05), and the DE and ME ranged from 13.77 MJ/kg to 17.05 MJ/kg and 13.40 MJ/kg to 16.73 MJ/kg, respectively. The DE and ME were negatively correlated to the NDF (*p* < 0.10) and ADF (*p* < 0.05) contents among the test cereals; however, the DE and ME contents were positively correlated with the starch content. Based on the chemical analysis, the models for predicting the DE and ME in the cereal feed ingredients were DE (MJ/kg) = 0.1364 × Starch (%) − 0.2352 × ADF (%) + 11.4467 and ME (MJ/kg) = 0.9838 × DE (MJ/kg) − 0.0165 × CP (%) + 0.0538. In conclusion, the different chemical compositions of different cereal feed ingredients led to large variations in the energy concentration and digestibility of nutrients for growing pigs. The number of cereal samples needs to be further increased to improve the accuracy of the equations for predicting energy contents.

## Simple Summary

It could be useful to rapidly and accurately evaluate the available energy in feed ingredients when formulating pig feed. Some researchers have studied equations for predicting the available energy for pigs using the chemical composition of a single cereal sample. Currently, many mixed feed ingredients are used to prepare pig feed. However, the development of equations to predict the energy contents for pigs when different kinds of cereal feed are mixed together has not been studied.

## 1. Introduction

Cereals are the main source used to provide energy in the dietary formulations for animals due to their high content of starch. The corn–soybean meal diet is a typical pig feed formula in China, of which corn accounts for about 50~65%. In recent years, the import of corn has increased to 20 million tonnes, second only to soybean imports, due to the rapid development of the feed industry. Therefore, the application of a diversified pig diet formulation is an important strategy for reducing the dietary inclusion levels of corn and soybean meal [1]. Many recent publications have reported the nutritional value and feeding effects when wheat, rice, sorghum and barley are used to replace corn in pig production [2,3]. There is some evidence that the moderate inclusion of wheat, rice, sorghum and barley to replace corn in pig diets has no negative effects on growth performance or nutrient digestibility [4,5,6,7].

An accurate evaluation of the available energy and standardized ileal amino acid digestiblity in wheat, rice, sorghum and barley is a prerequisite for their efficient utilization in pig production. The rapid detection of the available energy in and nutrient digestibility of feed ingredients is of great significance for accurate dietary formulation [8]. The digestibility of energy and nutrients among feed ingredients are influenced by the cereal cultivar, planting area and growth environment. Therefore, there are large variations in the nutritional values of various feed ingredients by country. There are some disadvantages to using animal digestion and metabolism tests to determine the available energy in feed ingredients with different sources and properties, such as their time-consuming nature, high costs and large errors [9]. One effective way to solve the above problems is establishing models to predict the available energy in feed ingredients by using conventional chemical compositions. To date, many studies on the determination and prediction of the available energy in and the nutrient digestibility of corn, wheat, barley and sorghum have been studied [10,11,12]. However, the studies mentioned above have only focused on developing available energy contents for a single cereal feed ingredient by using variations in chemical composition. Few studies have focused on determining the available energy based on the chemical compositions of multiple feed ingredients or have developed equations to predict this element. Therefore, this study was created to determine the available energy in and nutrient digestibility of 13 different cereal feed ingredients, including 5 corn samples, 2 wheat samples and 6 rice samples, and develop equations to predict the digestible (DE) and metabolizable energy (ME) based on their chemical compositions for growing pigs.

## 2. Materials and Methods

All the protocols used in this study were reviewed and approved by the Institutional Animal Care and Use Committee at China Agricultural University (CAU AW31604202-1-2). The experiment was conducted in the Metabolism Laboratory at the Swine Nutrition Research Centre of China Agricultural University (Chengde, Hebei Province, China). All samples of cereal were provided by New Hope Liuhe Co., Ltd. (Qingdao, Shandong Province, China).

### 2.1. Experimental Design

A total of 13 castrated boars with a body weight (BW) of 45.32 ± 1.45 kg were fed 13 diets formulated with 13 different cereal feed ingredients, including 5 corn samples, 2 wheat samples and 6 rice samples, according to a 13 × 6 Youden square design. The experiment included 6 periods, and one pig was fed one of the 13 diets in each period of 12 d (7 d for adapting diets and 5 d for total urine and feces collection) (Supplementary Table S1). Each dietary treatment included 6 replicates. The chemical compositions of 13 different cereal ingredients were determined (Table 1). The energy contents and nutrient digestibility for 5 samples of corn, 2 samples of wheat and 4 samples of rice were determined using the direct method, and those for 2 additional samples of rice were evaluated using the difference method, meaning that that the DE, ME and nutrient digestibility for the rice 5 and rice 6 samples were calculated according to the replacement proportion of tested feed ingredients in the corn 5 control diet (the calculation method is listed below). Samples of rice 5 and rice 6 were used to replace 40% of corn 5 (Table 2). Vitamins and minerals were supplemented in all the diets to meet or exceed the estimated nutrient requirements for growing pigs, as recommended by the NRC [13]. The chemical compositions of the experimental diets were determined (Table 3).

### 2.2. Feeding Management

The pigs were housed in the digestion and metabolism cages with a size of 1.5 m × 0.45 m × 0.6 m, and 12 cages were placed in the same feeding room with a controlled humidity of 55.0 ± 3.0% and a temperature of 22.0 ± 2.0 °C. Each cage was equipped with a feeder and water tank with a nipple drinker. Feed was provided at an amount of 4% of pig BW and offered to pigs at 0800 and 1600 h in each period of the experiment (Supplementary Table S2). The pigs drank water freely during the test period.

### 2.3. Sample Collection

Feces were collected every 6 h and immediately stored at −20 °C. A plastic bucket containing 30 mL of 6 N HCl was placed under each cage. Urine was collected once daily, and a 5% sub-sample was stored at −20 °C. A sub-sample of urine was collected for analysis. At the end of the experiment, the weight of all feces for each pig collected over 5 days were recorded, and two trays of about 350 g of feces were taken for drying with a temperature of 65 °C for 70 h by using the oven and then re-damped for 24 h. Samples of feces were crushed and passed through a 40-mesh sieve and bagged for further analysis. Samples of the urine for each pig were mixed and then filtered by using the gauze. About 10% of the urine was taken into a 50 mL centrifuge tube and stored at −20 °C.

### 2.4. Lab Analysis

Samples of test ingredients, diets and feces were collected by passing through a 1 mm screen. All samples above were analyzed for ash (Method 975.03), crude protein (CP; Method 990.03), dry matter (DM; Method 927.05), ether extract (EE; Method 920.39) and starch (Method 979.10), and neutral detergent fiber (NDF) and acid detergent fiber (ADF; method 973.18) were also analyzed [14]. Gross energy (GE) was determined using an Adiabatic Oxygen Bomb Calorimeter (Parr Instruments, Moline, IL, USA) with benzoic acid as the standard. About 4 mL of urine was used to filter paper and dried at 65 °C for 24 h to determine the GE. Samples of test ingredients, diets and feces were analyzed in duplicate, while the urinary energy was analyzed in triplicate to obtain accurate results.

### 2.5. Calculation

The energy content and the digestibility of nutrients in 5 samples of corn, 2 samples of wheat and 4 samples of rice were determined using the direct method, and those for 2 additional rice samples were evaluated using the difference method due to their high content of fiber components. The DE and ME contents of the 13 tested cereal samples were calculated as follows:

DE_d_ = ((GE_i_ − GE_f_)/FI)/0.974;
ME_d_ = ((GE_i_ − GE_f_ − GE_u_)/FI)/0.974;
DE_r_ = (DE_d_ − DE_c5_ × (1 − 0.40))/0.3896;
ME_r_ = (ME_d_ − ME_c5_ × (1 − 0.40))/0.3896;
ATTD_d_ = (N_d_ × FI − N_f_ × FO)/(N_d_ × FI);
ATTD_r_ = (N_d_ × ATTD_d_ − N_b_ ×ATTD_d_)/(N_r_ × X);

where GE_i_ represents the intake of GE for each pig (kcal, DM basis); FI represents the actual feed intake over the 5 d of the total feces and urine collection period; GE_f_ and GE_u_ represent the GE contents in the feces and urine for each pig (kcal, DM basis) over the 5 d of the collection period; DE_d_ and ME_d_ represent the DE and ME values for each diet (kcal/kg, DM basis); 0.974 represents the percentage of corn and soybean meal in the basal diet; DE_r_ and ME_r_ represent the DE and ME values for the rice 5 and rice 6 samples (kcal/kg, DM basis); 0.40 represents the proportion of rice 5 and rice 6 samples replacing the corn 5 in the diet; 0.3896 represents the percentage of rice 5 and rice 6 in the diets; DE_c5_ and ME_c5_ represent the DE and ME values for the corn 5 diet (kcal/kg, DM basis); ATTD_d_ (%) represents the total tract digestibility of the nutrients in the diet; N_d_ and N_f_ represents the nutrient concentrations in the diet and feces; FO is the output of feces; ATTD_c_ represents the total tract digestibility of the nutrients in the rice 5 and rice 6 samples; N_c5_ and Nr represent the nutrient concentrations in the corn 5 diet and tested rice 5 or rice 6 samples; and X represents the proportion of nutrients in rice 5 or rice 6 accounted for in the tested diets.

### 2.6. Statistical Analysis

The UNIVARIATE procedure (SAS 9.4, Cary, NC, USA) was performed to check normality and outliers. The pig in each cage was considered as the experimental unit. The diets were considered as the fixed effect, and the period was considered a random effect. The data were analyzed with a generalized linear model (GLM) using the mixed procedure. The results were shown as the mean ± SEM by the LSMEANS method, and significant differences among the diets were indicated using Tukey’s test. Correlations between available energy and the chemical compositions of the 13 cereal ingredients were analyzed by the PROC CORR procedure. The prediction equations for available energy were established according to the procedures of Stepwise Regression. Significant difference was considered when *p* < 0.05, while a tendency was considered when 0.05 ≤ *p* < 0.10.

## 3. Results

### 3.1. Chemical Analysis of 13 Cereal Feed Ingredients

The contents of GE, starch, CP, EE, NDF, ADF and ash (DM basis) ranged from about 17.72 MJ/kg to 21.20 MJ/kg, 37.53% to 71.40%, 7.88% to 16.25%, 1.30% to 16.12%, 6.74% to 27.65%, 1.60% to 14.80% and 1.18% to 5.55%, respectively, with CVs of 4.70%, 9.37%, 31.23%, 106.82%, 38.08%, 83.09% and 62.54% (Table 1).

### 3.2. Dietary Energy and Nutrient Digestibility

There were significant differences (*p* < 0.05) in the contents of DE and ME, the ME/DE ratio, and the nutrient digestibility among all the diets (Table 4).

### 3.3. Energy Content and Nutrient Digestibility of 13 Cereals

There were significant differences (*p* < 0.05) in the DE and ME contents, ME/DE ratio and nutrient digestibility among the 13 cereal feed ingredients (Table 5). The DE and ME ranged from about 13.77 MJ/kg to 17.05 MJ/kg DM, 13.40 MJ/kg to 16.73 MJ/kg DM and 96.59% to 99.01%, with the largest DE and ME being seen in the corn 1 sample and the lowest DE and ME being seen in the rice 6 sample. The digestibility of GE, DM, CP, NDF, ADF and ash were from 63.15% to 93.28%, 66.08% to 93.84%, 66.23% to 87.58%, 17.79% to 64.21%, 6.18% to 57.61% and 10.66% to 57.33%, respectively.

### 3.4. Correlated Analysis Between Energy Content and Chemical Analysis

The contents of the DE and ME were negatively correlated to the NDF (*p* < 0.10) and ADF (*p* < 0.05) contents in the cereal feed ingredients (Table 6) but positively correlated with the starch content. The ME of the cereal feed ingredients was positively correlated with the DE content (*p* < 0.05).

### 3.5. Equations for Predicting Available Energy in Cereal Feed Ingredients

Equations for predicting the DE and ME in cereal feed ingredients for growing pigs based on chemical composition were developed as the following (Table 7): DE (MJ/kg) = −0.1740 × ADF (%) + 14.3722 (*R*^2^ = 0.7899; *p* < 0.05) and 0.1364 × Starch (%) − 0.2352 × ADF (%) + 11.4467 (*R*^2^ = 0.8633; *p* < 0.05). The ME prediction equations were ME (MJ/kg) = 0.9716 × DE (MJ/kg) + 0.0636 (*R*^2^ = 0.9907; *p* < 0.05) and 0.9838 × DE (MJ/kg) − 0.0165 × CP (%) + 0.0538 (*R*^2^ = 0.9943; *p* < 0.05).

## 4. Discussion

There are some publications that have reported on the chemical compositions of different cereal samples and the variations in these compositions. Zhang et al. [15] evaluated 12 samples of corn, and the contents of GE, starch, CP, NDF, ADF and EE ranged from 18.19 MJ/kg to 18.62 MJ/kg, 71.41% to 74.42%, 8.13% to 9.31%, 10.72% to 15.34%, 2.05% to 2.97% and 3.33% to 5.53%, respectively; these results were consistent with our results for the chemical compositions of five samples of corn, except for the lower starch content in the present study. Their study analyzed 100 samples of corn and, in agreement with a previous study [11], showed that the contents of GE, starch, CP, NDF, ADF and EE were from 18.21 MJ/kg to 18.96 MJ/kg, 53.46% to 79.80%, 7.78% to 11.03%, 9.56% to 17.36%, 1.86% to 2.95% and 2.04% to 4.81%, respectively. Zhao et al. [10] reported that contents of GE, CP and starch among the 12 different wheat cultivars ranged from 18.33 MJ/kg to 18.63 MJ/kg, 12.95% to 18.14% and 60.81% to 70.77%, respectively. Another publication reported that the contents of GE, starch, CP, NDF, ADF and EE among 12 wheat samples with different planting areas ranged from 18.12 MJ/kg to 18.82 MJ/kg, 65.70% to 76.50%, 9.81% to 16.92%, 9.82% to 14.34%, 3.11% to 2.43%, 1.35% to 2.43% and 1.54% to 2.18%, respectively [16]. The above data on CP content were consistent with the chemical compositions of the wheat samples used in the present study, but higher GE and starch contents and lower NDF and ADF concentrations for the wheat samples in the previous report were observed compared with those for the wheat samples in our study. The high contents of NDF (19.51%) and ADF (3.62%) in the wheat 2 sample were consistent with previous studies [17,18,19]. In the present study, the contents of GE, starch, CP, NDF, ADF, EE and ash among the five samples of corn were from 18.22 MJ/kg to 18.58 MJ/kg, 64.78% to 68.34%, 8.11% to 12.63%, 7.18% to 13.33%, 1.60% to 3.42%, 2.74% to 4.35% and 1.18% to 1.79%, respectively. The corn 5 sample showed a high content of CP compared with the samples in the previous reports [11,15]. The two samples of wheat showed a similar chemical composition and GE content. The contents of GE, starch, CP, NDF, ADF, EE and ash in the six samples of rice ranged from 17.72 MJ/kg to 21.20 MJ/kg, 37.53% to 71.45%, 7.88% to 16.13%, 6.74% to 27.65%, 1.78% to 14.80%, 1.59% to 16.12% and 1.46% to 5.55%, respectively, which were consistent with the data reported by previous studies [20,21]. Surprisingly, the rice 3 sample showed the greatest contents of EE and CP, while the rice 1 sample showed the greatest starch content and a lower fiber concentration than the other rice samples. The above findings indicate that rice 3 could be mixed with oil according to the high EE content, while rice 1 could be polished and peeled to decrease the proportion of fiber components.

It should be noted that an imbalance or deficiency in CP and amino acids in a diet can affect energy utilization. However, it was difficult to maintain the balance between amino acids and adequate levels of them even though crystalline amino acids were provided in the diets. In the present study, the limitation for determining the DE and ME was that the test feed ingredient was the sole source of energy-supplied substrate in each diet, which would have led to an imbalance or deficiency in CP and amino acids for the pigs. According to the NRC [13], Yellow Dent corn has a DE of 16.39 MJ/ kg DM and an ME of 16.12 MJ/kg DM. Li et al. [11] found that the DE and ME ranged from 16.71 MJ/kg to 17.16 MJ/kg DM and 16.17 MJ/kg to 16.59 MJ/kg DM among 100 corn samples, respectively. In agreement with our findings, another publication reported that the DE and ME in four different varieties of corn ranged from 15.82 MJ/kg DM to 16.14 MJ/kg DM and 15.51 MJ/kg DM to 15.87 MJ/kg DM, respectively [22]. The data in the above publications were consistent with our results in the present study. A previous study reported that no interaction was observed between maize variety and storage duration regarding the DE and ME contents of corn but that the DE and ME contents of corn increased from 0 to 3 months and decreased from 3 to 10 months after storage [23]. Therefore, the large variations in the DE and ME contents of corn reported by the present study and other publications are related to the storage duration and drying methods as well as the varieties and planting areas [24]. The previous study also reported that the digestibility of GE, CP, NDF and ADF for four different varieties of corn ranged from 86.77% to 89.93%, 76.10% to 83.36%, 44.87% to 63.79% and 35.14% to 65.21%, respectively [22]. The digestibilities of nitrogen and GE in corn for growing pigs were 80% and 87%, respectively [25], and the above data on the nutrient digestibility of corn were consistent with our results for the nutrient digestibility of corn 5. The digestibility of GE and DM in the rice 1 sample is probably due to the high content of starch. Generally, the samples of wheat showed greater digestibility in terms of NDF and ADF for pigs compared with the samples of corn and rice, which could have been caused by the physical properties of fiber components derived from wheat, such as expansion.

The DE and ME contents and ME/DE ratio for 12 wheat samples of different cultivars for growing pigs ranged from 16.39 MJ/kg to 17.00 MJ/kg DM, 15.71 MJ/kg to 16.47 MJ/kg DM and 95.90% to 97.22%, respectively [10]. Similarly to the above publication, the other report indicated that the DE content of wheat cultivars ranged from 16.17 MJ/kg to 16.96 MJ/kg DM for five different classes of Canada western red spring wheat [26]. Therefore, the DE and ME contents for two samples of wheat in the present study were lower than those reported in the above publications but were similar to the data reported by the NRC [13]. The lower DE and ME contents than those in the previous reports could be due to the low starch contents of the five samples of corn used in the present study. Wiseman et al. [18] studied 16 wheat samples, collected from eight different varieties and two planting areas per variety, and indicated that the energy contents were affected by the variety and growth site based on the observation of large differences in the DE and ME contents among the test wheat samples. The previous study reported that the digestibility of GE ranged from 88.14% to 90.20% for 12 wheat samples [10], which was similar to our results showing that GE digestibility ranged from 86.07% to 90.27%.

A previous study reported the DE (14.70 MJ/kg and 14.88 MJ/kg, as-fed basis) and ME (14.31 MJ/kg and 14.22 MJ/kg) of brown rice stored for 1 and 6 years [27]. Dadalt et al. [28] showed that the DE, ME and ATTD of CP and GE for broken rice for weaned pigs were 15.67 MJ/kg DM, 15.57 MJ/kg, 77.89% and 90.60%, respectively. The reported data above were close to the results in the present study. The rice 5 and rice 6 samples had lower DE and ME contents, as well as lower nutrient digestibility, due to the high concentrations of fiber components. The lower nutrient digestibility of rice 3 may have been caused by the low digestibility of the fiber components compared with that of the other rice samples, indicating that the fiber components of rice 3 are primary insoluble fibers and are difficult for the gut microbiota to degrade [29]. However, the above hypothesis should be further verified by determining the lignin and cellulose contents.

Similarly to previous studies, the DE and ME showed negative correlations with the NDF and ADF contents and positive correlations with the starch content of the cereal feed ingredients in the present study. In addition, some previous studies reported positive correlations with EE, CP and GE but a negative correlation with ash in regard to the available energy contents in the cereals [30,31,32]; however, no such correlations were observed in our study. Many researchers have developed equations to predict the DE and ME for a single cereal feed ingredient, such as corn, wheat, barley and sorghum [33,34]. In a previous study [10], the best DE and ME prediction equations based on 12 different wheat cultivars were DE (Kcal/kg) = −2738 − (40.8 × ADF) + (1.7 × GE) − (51.5 × xylans) − (95.7 × Ash) + (22.3 × EE) and ME (kcal/kg) = −2990 + (1.7 × GE) − (50.2 × xylans) − (87.6 × Ash). The best DE and ME prediction equations for the corn were DE (Kcal/kg) = 1874 − (21.35 × NDF) + (0.65 × GE) − (99.84 × CF) and DE (kcal/kg) = 671.54 + (0.89 × DE) − (5.57 × NDF) − (191.39 × Ash) [11]. The suitable equations for predicting DE and ME based on three cereal grains and eight by-product ingredients were DE (kcal/kg) = 4055 − (3.09 × NDF) + (1.61 × CP) + (6.32 × EE) − (6.47 × ash) and ME (kcal/kg) = 3975 − (2.99 × NDF) + (1.38 × CP) + (6.62 × EE) − (7.45 × ash) [32]. However, there was no publication on equations for predicting available energy based on the chemical compositions of different types of cereal samples. Zhang et al. [35] developed an equation to predict the net energy for energy, protein and fiber feed ingredients for growing pigs based on recent published data. In the present study, the best DE and ME prediction equations determined using five corn, two wheat and six rice samples were DE (MJ/kg) = 0.1364 × Starch (%) − 0.2352 × ADF (%) + 11.4467 and ME (MJ/kg) = 0.9838 × DE (MJ/kg) − 0.0165 × CP (%) +0.0538. Importantly, extra cereal feed ingredients should be chosen to verify the accuracy of the above DE and ME prediction equations among the samples of cereal ingredients.

## 5. Conclusions

In conclusion, the varying chemical compositions of 13 different cereal feed ingredients lead to large variations in energy contents and digestibility in the nutrients for growing pigs. Equations for predicting energy content when different kinds of cereal feed are mixed together could be established. The best prediction equations for the DE and ME in the 13 samples of corn, wheat and rice were DE (MJ/kg) = 0.1364 × Starch (%) − 0.2352 × ADF (%) + 11.4467, and ME (MJ/kg) = 0.9838 × DE (MJ/kg) − 0.0165 × CP (%) +0.0538. The samples of cereal feed ingredients need to be further increased to improve the accuracy of the prediction equations.

## Figures and Tables

**Table 1:** Chemical compositions of 13 cereal feed ingredients (%, DM basis).

	Corn					Wheat		Rice						CV
Item	1	2	3	4	5	1	2	1	2	3	4	5	6	
DM	87.01	88.11	86.16	86.91	86.58	89.28	87.95	86.86	87.49	89.80	88.70	88.85	89.09	1.33
Ash	1.36	1.37	1.65	1.79	1.18	1.93	1.73	1.46	4.44	4.20	1.94	5.40	5.55	62.54
EE	3.62	4.35	2.74	3.01	2.97	1.34	1.30	2.75	1.59	16.12	3.58	2.02	1.74	106.82
NDF	12.94	11.62	13.33	11.55	7.18	14.84	19.51	6.74	16.01	18.19	18.04	22.23	27.65	38.08
ADF	3.42	2.80	3.25	2.31	1.60	3.50	3.62	1.78	10.78	7.61	3.95	14.76	14.80	83.09
CP	8.11	8.89	9.30	8.98	12.63	16.25	16.14	9.35	8.27	16.13	9.31	7.88	7.88	31.23
Starch	65.85	66.54	64.78	67.77	68.34	57.52	53.43	71.45	61.76	37.53	59.22	54.48	49.45	9.37
GE, MJ/kg	18.43	18.50	18.22	18.37	18.58	18.41	18.28	18.32	17.92	21.20	18.31	17.84	17.72	4.70

The chemical compositions of all the cereal feed ingredients were analyzed in duplicate. ADF, acid detergent fiber; CP, crude protein; DM, dry matter; EE, ether extract; GE, gross energy; NDF, neutral detergent fiber.

**Table 2:** Ingredient compositions of the diets (%).

	Corn Diets					Wheat Diets		Rice Diets ^2^					
Items	1	2	3	4	5	1	2	1	2	3	4	5	6
Corn 1	97.4	-	-	-	-	-	-	-	-	-	-	58.44	58.44
Corn 2	-	97.4	-	-	-	-	-	-	-	-	-	-	-
Corn 3	-	-	97.4	-	-	-	-	-	-	-	-	-	-
Corn 4	-	-	-	97.4	-	-	-	-	-	-	-	-	-
Corn 5	-	-	-	-	97.4	-	-	-	-	-	-	-	-
Wheat 1	-	-	-	-	-	97.4	-	-	-	-	-	-	-
Wheat 2	-	-	-		-	-	97.4	-	-	-	-	-	-
Rice 1	-	-	-	-	-	-	-	97.4	-	-	-	-	-
Rice 2	-	-	-	-	-	-	-	-	97.4	-	-	-	-
Rice 3	-	-	-	-	-	-	-	-	-	97.4	-	-	-
Rice 4	-	-	-	-	-	-	-	-	-	-	97.4	-	-
Rice 5	-	-	-	-	-	-	-	-	-	-	-	38.96	-
Rice 6	-	-	-	-	-	-	-	-	-	-	-	-	38.96
Dicalcium phosphate	0.9	0.9	0.9	0.9	0.9	0.9	0.9	0.9	0.9	0.9	0.9	0.9	0.9
Limestone	0.9	0.9	0.9	0.9	0.9	0.9	0.9	0.9	0.9	0.9	0.9	0.9	0.9
NaCl	0.3	0.3	0.3	0.3	0.3	0.3	0.3	0.3	0.3	0.3	0.3	0.3	0.3
Premix ^1^	0.5	0.5	0.5	0.5	0.5	0.5	0.5	0.5	0.5	0.5	0.5	0.5	0.5
Total	100	100	100	100	100	100	100	100	100	100	100	100	100

^1^ Premix provided the following per kg of diet for growing pigs: 5000 IU of vitamin A; 2000 IU of vitamin D_3_; 25 IU of vitamin E; 2.0 mg of vitamin K_3_; 25.0 μg of vitamin B_12_; 5.0 mg of riboflavin; 15.0 mg of pantothenic acid; 28.0 mg of niacin; 420.0 mg of choline chloride; 0.8 mg of folacin; 1.6 mg of thiamine; 3.5 mg of pyridoxine; 50.0 μg of biotin; 45.0 mg of Mn; 75.0 mg of Fe; 50.0 mg of Zn; 80.0 mg of Cu; 0.4 mg of I; 0.4 mg of Se. ^2^ The rice 5 and rice 6 diets were formulated using the sample of corn 5.

**Table 3:** Chemical compositions of the diets (%, DM basis).

	Corn					Wheat		Rice					
Item	1	2	3	4	5	1	2	1	2	3	4	5	6
DM	89.33	88.22	87.00	87.57	86.66	88.10	88.88	87.82	88.49	90.98	88.56	87.88	87.15
Ash	3.77	3.46	3.99	4.12	3.45	3.99	4.04	3.58	5.88	10.10	3.98	5.11	4.33
EE	3.82	2.40	0.82	2.85	3.01	0.87	1.09	1.29	0.98	15.47	2.74	2.54	2.13
NDF	12.91	8.80	12.72	12.20	9.85	15.83	14.79	5.91	16.52	17.91	13.24	13.91	15.31
ADF	3.49	2.45	2.03	3.08	2.76	4.07	3.91	1.89	10.67	8.00	3.80	7.11	7.95
CP	8.21	8.86	9.14	9.27	9.66	15.24	15.31	9.15	7.63	15.30	8.26	9.37	8.96
Starch	64.24	69.25	66.57	64.27	67.32	57.34	57.85	73.42	61.58	34.43	64.67	62.43	62.75
GE, MJ/kg	18.55	17.68	17.79	17.88	18.10	17.41	17.70	17.32	17.03	20.54	17.88	17.58	17.71

The chemical compositions of the diets were analyzed in duplicate. ADF, acid detergent fiber; CP, crude protein; DM, dry matter; EE, ether extract; GE, gross energy; NDF, neutral detergent fiber.

**Table 4:** The DE, ME and digestibility of nutrients in the diets for growing pigs.

	Corn					Wheat		Rice							
Item	1	2	3	4	5	1	2	1	2	3	4	5	6	SEM	*p*-Value
As-fed basis															
DE, MJ/kg	14.45 ^a^	14.08 ^abc^	13.32 ^def^	13.62 ^cd^	13.76 ^bcd^	13.44 ^de^	13.71 ^cd^	14.19 ^ab^	12.35 ^g^	13.55 ^d^	14.37 ^a^	13.07 ^ef^	12.94 ^f^	0.07	<0.01
ME, MJ/kg	14.72 ^a^	13.75 ^abc^	12.99 ^def^	13.26 ^cde^	13.47 ^bcd^	13.09 ^def^	13.25 ^cde^	13.87 ^ab^	12.23 ^g^	13.14 ^def^	14.08 ^a^	12.81 ^ef^	12.66 ^fg^	0.08	<0.01
DM basis															
DE, MJ/kg	16.18 ^a^	15.96 ^ab^	15.31 ^cde^	15.55^bc^	15.88 ^ab^	15.25 ^cde^	15.42 ^bcd^	16.16 ^a^	13.96 ^f^	14.89 ^de^	16.23 ^a^	14.87 ^e^	14.75 ^e^	0.08	<0.01
ME MJ/kg	15.87 ^a^	15.59 ^ab^	14.93 ^cd^	15.14 ^bc^	15.54 ^ab^	14.85 ^cd^	14.90 ^cd^	15.80 ^a^	13.82 ^e^	14.44 ^d^	15.90 ^a^	14.57 ^cd^	14.53 ^d^	0.08	<0.01
ME, /DE	98.09 ^ab^	97.66 ^ab^	97.50 ^ab^	97.36 ^ab^	97.87 ^ab^	97.40 ^ab^	96.59 ^b^	97.75 ^ab^	99.01 ^a^	96.96 ^b^	97.98 ^ab^	98.00 ^ab^	97.80 ^ab^	0.11	<0.01
Apparent total tract digestibility, %															
GE	87.77 ^bcd^	90.27 ^abc^	86.07 ^def^	86.96 ^de^	87.72 ^bcd^	87.58 ^cde^	87.17 ^de^	93.28 ^a^	81.96 ^g^	72.5 ^h^	90.75 ^ab^	84.57 ^efg^	83.83 ^fg^	0.58	<0.01
DM	88.43 ^bc^	91.16 ^ab^	87.52 ^c^	87.90 ^c^	88.58 ^bc^	88.54 ^bc^	87.47 ^c^	93.83 ^a^	81.77 ^d^	67.38 ^e^	90.32 ^bc^	83.86 ^d^	83.47 ^d^	0.73	<0.01
Ash	45.83 ^abc^	50.12 ^ab^	53.86 ^a^	46.04 ^abc^	41.99 ^abcd^	48.39 ^ab^	36.87 ^bcde^	57.33 ^a^	35.24 ^bcde^	22.50 ^e^	30.52 ^cde^	27.35 ^de^	22.51 ^e^	1.55	<0.01
NDF	55.09 ^ab^	54.12 ^ab^	50.96 ^b^	56.32 ^ab^	54.82 ^ab^	64.21 ^a^	56.78 ^ab^	58.91 ^ab^	25.61 ^cd^	17.79 ^d^	63.27 ^ab^	28.10 ^cd^	31.24 ^c^	1.86	<0.01
ADF	50.39 ^a^	47.27 ^a^	9.31 ^cd^	49.78 ^a^	57.60 ^a^	40.74 ^ab^	25.80 ^bc^	40.70 ^ab^	15.60 ^cd^	7.31 ^d^	13.01 ^cd^	11.97 ^cd^	17.35 ^cd^	2.22	<0.01
CP	76.36 ^de^	83.01 ^abcd^	73.89 ^e^	77.70 ^cde^	78.48 ^cde^	87.58 ^a^	83.63 ^abc^	86.81 ^ab^	77.19 ^cde^	66.23 ^f^	80.33 ^bcde^	78.32 ^cde^	78.38 ^cde^	0.71	<0.01

In the same row, values with different small letter superscripts are significantly different (*p* < 0.05), while those with the same letter or no superscripts are not significantly different (*p* > 0.05). n = 6. ADF, acid detergent fiber; CP, crude protein; DM, dry matter; EE, ether extract; GE, gross energy; NDF, neutral detergent fiber; SEM, standard error of the mean.

**Table 5:** The DE, ME and nutrient digestibility of 13 cereal feed ingredients.

	Corn					Wheat		Rice							
Item	1	2	3	4	5	1	2	1	2	3	4	5	6	SEM	*p*-Value
As-fed basis															
DE, MJ/kg	14.84 ^a^	14.45 ^abc^	13.68 ^def^	13.98 ^abc^	14.13 ^cd^	13.79 ^def^	14.08 ^cd^	14.57 ^ab^	12.68 ^g^	13.91 ^d^	14.75 ^a^	13.42 ^ef^	13.29 ^f^	0.13	<0.01
ME, MJ/kg	14.55 ^a^	14.12 ^abc^	13.34 ^def^	13.61 ^cde^	13.83 ^bcd^	13.43 ^def^	13.60 ^cde^	14.24 ^ab^	12.56 ^g^	13.49 ^def^	14.46 ^a^	13.15 ^ef^	13.00 ^fg^	0.15	<0.01
DM basis															
DE, MJ/kg	17.05 ^a^	16.40 ^bc^	15.88 ^de^	16.09 ^bcd^	16.75 ^ab^	15.45 ^de^	16.00 ^cd^	16.78 ^ab^	14.49 ^f^	15.49 ^de^	16.63 ^ab^	14.18 ^fg^	13.77 ^g^	0.16	<0.01
ME, MJ/kg	16.73 ^a^	16.02 ^bc^	15.48 ^de^	15.67 ^bcd^	16.40 ^ab^	15.05 ^de^	15.46 ^cd^	16.40 ^ab^	14.35 ^f^	15.02 ^de^	16.30 ^ab^	13.87 ^fg^	13.40 ^g^	0.17	<0.01
ME/DE	98.10 ^ab^	97.66 ^b^	97.50 ^bc^	97.37 ^bc^	97.88 ^b^	97.39 ^bc^	96.59 ^c^	97.75 ^b^	99.01 ^a^	96.96 ^bc^	97.98 ^ab^	97.84 ^b^	97.30	0.21	<0.01
Apparent total tract digestibility, %															
GE	87.77	90.27 ^abc^	86.07 ^d^	86.96 ^d^	87.72 ^bcd^	87.58 ^cd^	87.17 ^d^	93.28 ^a^	81.96 ^e^	72.50 ^f^	90.75 ^ab^	69.10 ^f^	63.15 ^g^	0.83	<0.01
DM	88.43 ^bc^	91.15 ^ab^	87.52 ^c^	87.90 ^c^	88.58 ^bc^	88.54 ^bc^	87.47 ^c^	93.84 ^a^	81.77 ^d^	67.38 ^f^	90.32 ^bc^	74.67 ^e^	66.08 ^f^	0.99	<0.01
Ash	45.83 ^abc^	50.12 ^ab^	53.86 ^a^	46.04 ^abc^	41.99 ^abcd^	48.39 ^ab^	36.87 ^bcde^	57.33 ^a^	35.24 ^bcde^	22.50 ^e^	30.52 ^cde^	10.66 ^f^	16.38 ^ef^	2.23	<0.01
NDF	55.09 ^ab^	54.12 ^ab^	50.96 ^a^	56.32 ^ab^	54.82 ^ab^	64.21 ^a^	56.78 ^ab^	58.91 ^ab^	25.61 ^d^	17.79 ^d^	63.27 ^ab^	57.48 ^ab^	40.69 ^c^	2.28	<0.01
ADF	50.39 ^a^	47.27 ^a^	9.31 ^cd^	49.78 ^a^	57.60 ^a^	40.74 ^ab^	25.80 ^bc^	40.70 ^ab^	15.60 ^cd^	7.31 ^d^	13.01 ^cd^	35.99 ^ab^	39.44 ^ab^	2.53	<0.01
CP	76.36 ^de^	83.01 ^ab^	73.89 ^e^	77.70 ^cde^	78.48 ^cde^	87.58 ^a^	83.63 ^abc^	86.81 ^ab^	77.19 ^cde^	66.23 ^f^	80.32 ^bcde^	86.40 ^ab^	80.77 ^cd^	1.01	<0.01

In the same row, values with different small letter superscripts are significantly different (*p* < 0.05), while those with the same letter or no superscripts are not significantly different (*p* > 0.05). n = 6. ADF, acid detergent fiber; CP, crude protein; DM, dry matter; EE, ether extract; GE, gross energy; NDF, neutral detergent fiber; SEM, standard error of the mean.

**Table 6:** Correlated analysis between chemical analysis of and energy contents in 13 test ingredients.

Item	Dry Matter	Ash	Ether Extract	Neutral Detergent Fiber	Acid Detergent Fiber	Crude Protein	Starch	Gross Energy	Digestible Energy	Metabolizable Energy
Dry matter	1.00									
Ash	0.68	1.00								
Ether extract	0.41	0.64	1.00							
Neutral detergent fiber	0.70	0.63	0.05	1.00						
Acid detergent fiber	0.54	0.75 *	0.01	0.81 **	1.00					
Crude protein	0.40	0.16	0.39	0.02	−0.26	1.00				
Starch	0.75 *	023	−0.46	−0.32	−0.23	0.32	1.00			
Gross energy	0.58	0.63	0.95	0.11	0.00	0.62	0.43	1.00		
Digestible energy	−0.34	−0.60	0.17	−0.69 *	−0.89 **	0.20	0.84 **	0.17	1.00	
Metabolizable energy	−0.37	−0.62	0.15	−0.71 *	−0.88 **	0.14	0.81 **	0.14	0.99 **	1.00

** *p* < 0.05, * 0.05 ≤ *p* < 0.10. The results of the correlation analysis were obtained based on the mean values of the DE, ME and chemical composition of the cereal feed ingredients.

**Table 7:** Equations for predicting DE and ME contents in cereal feed ingredients for growing pigs.

Item	Prediction Equation	*R* ^2^	*p*-Value
1	DE (MJ/kg) = −0.1740 × ADF (%) + 14.3722	0.7899	<0.01
2	DE (MJ/kg) = 0.1364 × Starch (%) − 0.2352 × ADF (%) + 11.4467	0.8633	<0.01
3	ME (MJ/kg) = 0.9716 × DE (MJ/kg) + 0.0636	0.9907	<0.01
4	ME (MJ/kg) = 0.9838 × DE (MJ/kg) − 0.0165 × CP (%) + 0.0538	0.9943	<0.01

The equations were developed based on the chemical compositions (as-fed basis) of 13 cereals. The coefficient of determination (*R*^2^) was used to determine the best prediction equation, and the largest *R*^2^ value was taken to indicate the best prediction model. ADF, acid detergent fiber; CP, crude protein; DE, digestible energy; ME, metabolizable energy.

## Data Availability

The raw data are available within this manuscript.

## Supplementary Materials

The following supporting information can be downloaded at: https://www.mdpi.com/article/10.3390/vetsci11120648/s1, Table S1: The operation of experimental design and animal grouping. Table S2: Change in pig body weight (BW, kg) and feed intake (FI, kg/d) among six different periods of the experiment.
