# Supplementary material for: Determination and Prediction of Available Energy in 13 Cereal Feed Ingredients for Growing Pigs
Source: Vet Sci. 2024 Dec 13;11(12):648. doi: 10.3390/vetsci11120648 (PMC11680150; doi:10.3390/vetsci11120648)
Supplement: Supplementary file 1 [file vetsci-11-00648-s001.zip › vetsci-3253651-supplementary.pdf]

veterinary sciences

MDPI

Supplementary Table S1. The operation of experimental design and animal grouping.

Pig Number	Period 1	Period 2	Period 3	Period 4	Period 5	Period 6
1	Diet 1	Diet 13	Diet 12	Diet 11	Diet 10	Diet 9
2	Diet 2	Diet 1	Diet 13	Diet 12	Diet 11	Diet 10
3	Diet 3	Diet 2	Diet 1	Diet 13	Diet 12	Diet 11
4	Diet 4	Diet 3	Diet 2	Diet 1	Diet 13	Diet 12
5	Diet 5	Diet 4	Diet 3	Diet 2	Diet 1	Diet 13
6	Diet 6	Diet 5	Diet 4	Diet 3	Diet 2	Diet 1
7	Diet 7	Diet 6	Diet 5	Diet 4	Diet 3	Diet 2
8	Diet 8	Diet 7	Diet 6	Diet 5	Diet 4	Diet 3
9	Diet 9	Diet 8	Diet 7	Diet 6	Diet 5	Diet 4
10	Diet 10	Diet 9	Diet 8	Diet 7	Diet 6	Diet 5
11	Diet 11	Diet 10	Diet 9	Diet 8	Diet 7	Diet 6
12	Diet 12	Diet 11	Diet 10	Diet 9	Diet 8	Diet 7
13	Diet 13	Diet 12	Diet 11	Diet 10	Diet 9	Diet 8

Supplementary Table S2. Change of pig body weight (BW, kg) and feed intake (FI, kg/d) among 6 different periods of the experiment.

Pig NO.	Period 1		Period 2		Period 3		Period 4		Period 5		Period 6	
	BW	FI	BW	FI	BW	FI	BW	FI	BW	FI	BW	FI
1	47.43	1.90	51.42	2.06	56.43	2.26	62.21	2.49	68.54	2.74	74.66	2.99
2	46.83	1.87	50.73	2.03	56.73	2.27	61.75	2.47	67.56	2.70	75.01	3.00
3	44.24	1.77	48.32	1.93	53.2	2.13	58.4	2.34	65.34	2.61	70.43	2.82
4	45.68	1.83	50.32	2.01	55.43	2.22	62.43	2.50	66.83	2.67	74.1	2.96
5	46.46	1.86	50.12	2.00	53.83	2.15	61.32	2.45	68.34	2.73	75.32	3.01
6	47.32	1.89	49.45	1.98	54.71	2.19	59.34	2.37	65.42	2.62	71.23	2.85
7	45.32	1.81	49.2	1.97	55.15	2.21	61.43	2.46	69.34	2.77	74.54	2.98
8	43.67	1.75	46.72	1.87	49.43	1.98	54.87	2.19	62.83	2.51	68.2	2.73
9	43.59	1.74	48.93	1.96	53.37	2.13	59.24	2.37	64.63	2.59	70.43	2.82
10	44.78	1.79	49.45	1.98	53.22	2.13	58.85	2.35	66.72	2.67	70.4	2.82
11	45.61	1.82	50.11	2.00	55.43	2.22	62.43	2.50	69.43	2.78	73.56	2.94
12	45.24	1.81	48.54	1.94	52.4	2.10	59.34	2.37	65.32	2.61	69.86	2.79
13	43.01	1.72	48.92	1.96	53.11	2.12	59.99	2.40	67.02	2.68	71.53	2.86
